# Resting time before slaughter restores homeostasis, increases rigor mortis time and fillet quality of surubim *Pseudoplatystoma* spp.

**DOI:** 10.1371/journal.pone.0233636

**Published:** 2020-05-22

**Authors:** Letícia Emiliani Fantini, Robson Andrade Rodrigues, Claucia Aparecida Honorato, Elenice Souza dos Reis Goes, André Luiz Julien Ferraz, Jorge Antonio Ferreira de Lara, Terry Hanson, Cristiane Meldau de Campos

**Affiliations:** 1 Animal Science Graduate Program, State University of Mato Grosso do Sul—UEMS, Aquidauana, Mato Grosso do Sul, Brazil; 2 Aquaculture Department, Federal University of Santa Catarina, Florianopolis—UFSC, Florianopolis, Santa Catarina, Brazil; 3 Animal Science Graduate Program, Federal University of Grande Dourados—UFGD, Dourados, Mato Grosso do Sul, Brazil; 4 Brazilian Agricultural Research Corporation, EMBRAPA Pantanal, Corumbá, Mato Grosso do Sul, Brazil; 5 School of Fisheries, Aquaculture and Aquatic Science, Auburn University (SFAAS-AU), Auburn, AL, United States of America; 6 Animal Science Graduate Program, Faculty of Veterinary Medicine and Animal Science, Federal University of Mato Grosso do Sul—UFMS, Campo Grande, Brazil; University of Messina, ITALY

## Abstract

This study assesses the respiratory dynamics related to stress parameters and resting time before slaughter, in the quality of surubim (*Pseudopatystoma* spp.) fillets. A completely randomized design was conducted using five treatments: resting time before slaughter of 0, 2, 4, 8 and 24 hours, with 15 fish sampled per treatment. Time 0 corresponded to the treatment without resting time, where the fish were slaughtered immediately after arriving at the processing plant. The resting time did not affect the electrolyte balance, hemoglobin, plasma, hepatic glycogen, myofibrillar fragmentation index (MFI) and water holding capacity (WHC) of surubins. However, with increased resting time, there was a significant decrease in muscle glycogen and an increase in blood pH and blood bicarbonate levels. Additionally, respiratory parameters showed an increase in pO_2_ and, consequently, in O_2_ saturation and a decrease in pCO_2_.The hematocrit and MCV values of the surubins after 24 hours of resting decreased significantly. In the first hours of resting, the highest values of erythrocytes and CHCM were observed. The lowest level of stress was observed for fish having 24 hours of resting. Fish having longer resting periods (8 and 24 hours) presented fillets with a higher pH (P <0.05) and the rigor mortis establishment time was shorter for the first 2 hours and 24 hours of resting time. There was a linear decrease in fillet lightness and an increase in the intensity of red (CIE a*) color up to 24 hours when resting was increased. In CIE b*, a linear decrease (P <0.05) of the yellow intensity of the fillets was observed as the surubim resting time increased. A resting time of 4 to 8 hours before slaughter is effective in reestablishing homeostasis after transporting surubim, providing fillets with higher quality and a greater length of the pre-rigor mortis period.

## Introduction

In aquaculture, the procedures involved in pre-slaughter management are recognized as a critical point in the management of fish welfare [1] and have important effects on meat quality [2]; [3]. In the pre-slaughter period, fish are stocked at high densities and activities associated with harvest, crowding and transport to the processing plant can result in stress from increased physical activity [2]; [4].

The excess activity that occurs during fish handling can promote changes in the respiratory responses [5], causing secondary adverse reactions such as acidosis and osmotic stress due to respiratory arrest and insufficient gas and ion exchange between blood and water [6]. Physiological factors such as increased plasma cortisol, glucose and chloride levels are used to determine the degree of stress in animals [7]. Additionally, stress can make the metabolism more anaerobic, which results in a lower glycogen content, leading to a faster decrease in pH and the early onset of rigor mortis [8]. This is a harmful factor for the industry, since filleting can only be carried out successfully when the fish is in pre- or post-rigor condition [9].

The onset and the strength of rigor mortis affect the fillet quality, due to faster autolysis and greater ruptures in connective and muscle tissues [10], factors that lead to the incidences of gaping [11], changes in color [12], juiciness [13], softness [4] and reduced water holding capacity [14]; [15], thus reducing the shelf life of the product [14]; [16].

Most studies of the metabolic responses of fish to intensive management practices in aquaculture do not cover surubim (*Pseudoplatystoma* spp.) The average carcass yield of surubim (*Pseudoplatystoma* ssp.) is 69.93 ± 1.61% [17], which characterizes this fish as being of great economic importance for the aquaculture industry [18]. Therefore, this study aimed at assessing the respiratory dynamics related to the parameters of stress and time of rest before slaughter to the instrumental measurements of surubim fillet (*Pseudopatystoma* spp.) quality.

## Material and methods

This experiment was carried out in accordance with the guidelines of the Brazilian College for Animal Experimentation (COBEA; http://www.cobea.org.br) and was approved by the Committee on Animal Care of the State University of Mato Grosso do Sul (009/2013UEMS)/Brazil.

### Experimental design

Specimens of surubim hybrids *(P*. *reticulatum* × *P*. *corruscans*) were used with an average weight of 1.09 ± 0.19 kg, raised in a 3.94 ha pond at a density of 4,000 kg ha^-1^, in Itaporã, Mato Grosso do Sul (22˚08 'S, 054˚47' W). Before being harvested and transported to the processing plant, the fish were fasted for 48 hours in order to have their digestive tract emptied [19]; [20].

The fish were transported for twenty minutes in four tanks of 2400 L (live hauling truck) with density of 415 kg m^3^ equipped with constant aeration to the processing plant. The water temperature was lowered with ice keeping it above 22 ºC. The fish were then unloaded into one 25 m^3^ tank with maximum fish density of 160 kg^-1^ m^3^. The tank was refilled with well water from a single reserve and aeration kept the dissolved oxygen level at 6 mg^-1^ L, pH at 6.8 and temperature of 28°C.

A completely randomized design was conducted, with five treatments: 0, 2, 4, 8 and 24 hours of resting time, with 15 fish sampled per treatment. Time 0 corresponded to the treatment with no resting time, where the fish were slaughtered immediately after arriving at the processing unit in accordance with processing plant protocol. From the 15 fish sampled per treatment, 10 specimens were used for the stress parameters, hematological, gas and fillet analyses. The fish were submitted to blood collection by puncture of the caudal vessel using heparinized syringes, always performed by the same trained operator. Blood aliquots were centrifuged at 1500 x g for five minutes for plasma separation, and then stored in liquid nitrogen. An average of 2 g of liver and white muscle (dorsal muscle) samples were also collected and immediately frozen in liquid nitrogen as well.

After blood collection, the fish were euthanized by a spinal cord section and placed in water and ice in a 1:1 ratio. Fish were filleted by hand, packaged into plastic bag, placed in a cooler with dry ice, and transported to the laboratory (Center of Agricultural Research of the Pantanal—EMBRAPA), an 8 hours trip, for meat quality analysis. The remaining 5 fish were kept whole (not gutted) and used only for rigor mortis analysis.

### Blood gas analysis and plasmatic ions

Blood samples were analyzed for pH, partial pressures of oxygen (pO_2_) and carbon dioxide (pCO_2_), bicarbonate (HCO^-^_3_), sodium (Na^+^), potassium (K^+^), Calcium (Ca) and chloride (Cl) according to [21].

### Hematological analysis

Hematocrit percentages were determined by the microhematocrit method by [22], hemoglobin [23], number of erythrocytes in Neubauer chamber and hematimetric indices: mean corpuscular volume (MCV) and mean corpuscular hemoglobin concentration (MCHC) [24].

### Stress parameters

Plasma aliquots were subjected to colorimetric glucose measurements [21] and cortisol was quantified by radioimmunoassay (RIA). Liver (50 mg) and muscle tissues (10 mg) were solubilized with 6.0 N KOH in a boiling water bath for 5 min, and the glycogen was precipitated by ethanol and K2SO4 saturated solution. After centrifugation at 2000 x g for 3 min, the pellets were resuspended in distilled water and the glycogen was quantified as glucosyl-glucose by phenol-sulfuric acid (Dubois et al., 1956). Glycogen was assayed in alcoholic precipitates from alkaline homogenates (Bidinotto et al., 1997) in spectrophotometer (Biospectro, model SP- 220) reading at 480 nm and expressed as μmol g of tissue ^-1^.

### Rigor mortis index

Rigor-mortis index (Ir) was carried out on an automatic system temperature-controlled room between 12–14°C. Five fish specimens from each treatment, immediately after slaughter and every 30 minutes for 4 hours, in order to identify the behavior of this phenomenon until its full installation (Ir = 100%). The Ir was determined according to the formula adapted from [25]. Ir = [(L0 − Lt) / L0] x 100. Where, L represents the vertical drop (cm) of the tail, when half of the fish fork length is placed beyond the edge of a table. L0 is the lowest measured drop of the tail fin, while Lt represents measurements throughout the experiment.

### Fillet instrumental analysis

**Fig 1:**
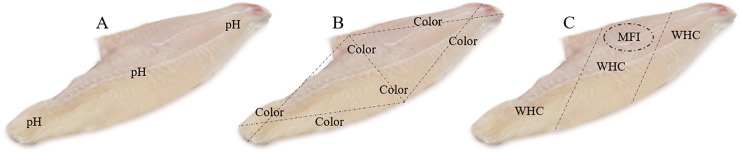
Surubin’s fillet meat quality was analyzed following the picture described above. The pH was taken in three points using a Mettler Sentron (model 1001) (A). Color (CIE L*, a*, b*) was determined at 6 points using a Minolta Camera (model CR-10, Ltd, Osaka, Japan) at an angle of 90° (B). Water holding capacity (WHC) was taken in three points on each fillet (C) using methodology described by (Nakamura and Katoh, 1985). Myofibril fragmentation index (MFI) was taken according to the methodology described by Culler et al. (1978) (C).

The following parameters were evaluated: pH (Fig 1A), color (Fig 1B), water holding capacity (WHC) (Fig 1C), and myofibril fragmentation index (MFI) (Fig 1C). For each parameter evaluated, three unique points were sampled on each fillet except for color, which was measured in six points.

The parameters pH and color were measured 24 hours after slaughter. The pH meter Sentron with a specific electrode for meat was used (model 1001). Color values (Hunter system) of CIE L* (lightness), CIE a* (redness), and CIE b* (yellowness) of the fillets were assessed using a colorimeter Minolta Camera (model CR-10, Ltd, Osaka, Japan), at an angle of 90°.

Water holding capacity was evaluated 48 hours after fillet processing (WHC) and was estimated [26] by centrifuging (in a 5430R Eppendorf centrifuge North America, Inc., New York, USA) 1 g of raw fillet placed on tissue paper inside a tube for 4 min at 1500 x g. The water remaining after centrifugation was quantified by drying the samples at 60°C for 12 hours. WHC was calculated as: (weight after centrifugation (g)–weight after drying (g))/initial weight (g) × 100.

For MFI the samples were frozen at -18°C and then thawed at a temperature of 7°C in the refrigerator for about 12 hours for analysis according to the methodology described by [27]. Protein concentration was determined by the Biuret method [28] readings were performed in a spectrophotometer, absorbance was measured immediately at 540 nm and the result was multiplied by 200 to give an MFI for each fillet.

### Statistical analysis

The results were submitted to the normality test and to the homogeneity test of the variances. For variables whose distribution was normal, the analysis of variance was applied and when significant, the means were compared by the Tukey test at 5% probability.

## Results

The resting time did not change the electrolyte (plasmatic ions) balance of the surubim submitted to different resting times (Table 1). The different resting times resulted in an increase in blood pH level along with blood bicarbonate levels (Table 1). Respiratory parameters showed an increase in pO_2_ and, consequently, in O_2_ saturation and a decrease in pCO_2_.

**Table 1:** Mean ± SE blood plasmatic ions, respiratory parameters and blood count of surubim submitted to a different resting time in a holding tank before slaughter.

	Resting time in a holding tank (hours)				
Parameters	0	2	4	8	24
**Plasmatic ions (nmol.L-**^**1**^**)**					
Sodium	152.4 ±2.6	144.9±2.0	147.6±4.1	137.9±3.9	144.4±4.4
Potassium	7.8 ±1.1	7.6±1.1	7.4±0.9	7.7±1.2	7.1±0.6
Calcium	1.0 ±0.1	1.1±0.1	0.9±0.3	0.9±0.1	1.0±0.2
Choride	122.7 ±2.0	115.3±2.0	118.6±3.9	111.1±2.3	110.4±3.2
**Respiratory parameters**					
pH	6.69 ±0.07^b^	6.76±0.05 ^b^	6.82±0.05 ^b^	6.33±0.02 ^b^	7.02±0.04^a^
pO_2_	37.47 ±2.72	37.13±4.36	38.2±5.16	45.92±1.32	47.34±5.72
pCO_2_	49.07 ±6.75	34.58±4.45	36.00±6.41	30.38±8.06	33.08±3.99
HCO_3_	5.86±0.42^b^	4.81±0.55 ^b^	5.71±0.90 ^b^	6.63±0.21^a^	8.29±0.95^a^
**Blood Count**					
Hematocrit (%)*	61.58 ± 0.85^a*^	63.05 ± 0.31^a*^	63.91 ± 0.55^a^	60.29±0.71^ab^	56.68 ± 0.88^b^
Erythrocytes (x10^6^ μL^-1^)*	4.84 ± 0.06^ab^	4.91 ± 0.10^ab^	5.24 ± 0.15^a^	4.90±0.19^ab^	4.56 ± 0.24^b^
Hemoglobin (g.dL^-1^)	24.05 ± 0.62**	23.02 ± 0.30**	25.06 ± 0.65*	23.99±0.59	22.92 ± 0.52*
MCHC (g.dL^-1^)*	39.01 ± 0.64^a*^	36.86 ± 0.26^b*^	40.26 ± 0.54^a^	40.33±0.47^a^	40.40 ± 0.40^a^
MCV (fL)*	129.90 ± 1.84^a*^	126.07 ± 0.76^ab^	117.68 ± 2.64^b^	118.14±1.69^b^	118.60 ± 0.74^b^

Means in the same row with different letters indicate statistical difference by the Tukey test (P<0.05). MCHC: mean corpuscular hemoglobin concentration. MCV: mean corpuscular volume.

The hematocrit and MCV values of the surubins after 24 hours of resting time showed a significant decrease. Hemoglobin did not respond to the resting time. In the first hours of rest, the highest values of erythrocytes and MCHC were observed.

**Fig 2:**
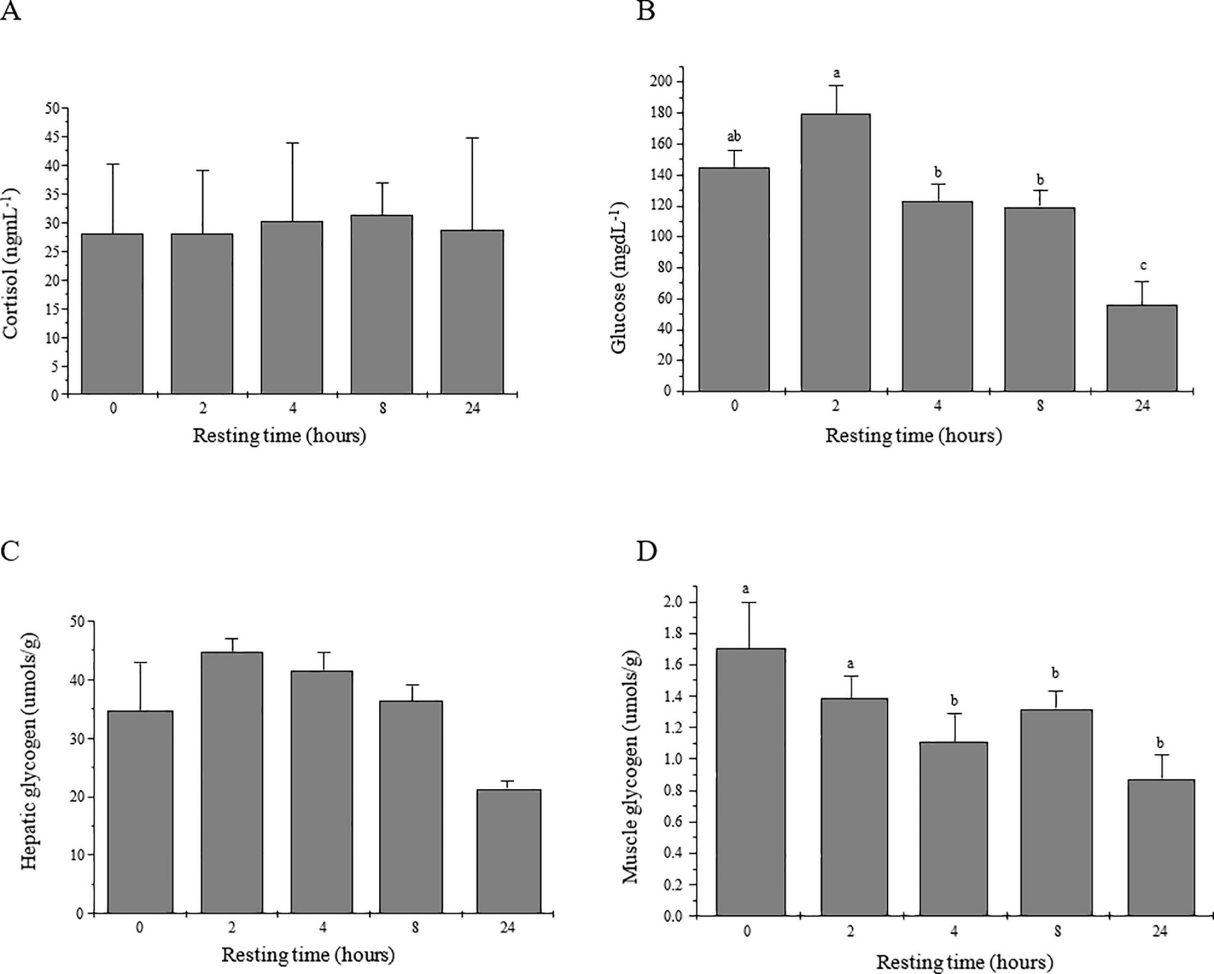
Mean values (± SE) of cortisol (ngmL^-1^) (A) plasma glucose (mgdL^-1^) (B), hepatic glycogen (C) and muscle glycogen (D) (μmols/g/tissue) of surubim *Pseudoplatystoma* spp. submitted to different resting times in a holding tank before slaughter. Different letters indicate statistical difference (P <0.05) by the Tukey test.

There was no difference for plasma cortisol levels among resting times (Fig 2A). For plasma glucose levels, lower levels were observed after 4 hours of rest with the lowest level observed for fish submitted to 24 hours of rest (Fig 2B). There was no decrease in hepatic glycogen (Fig 2C), however, there was a significant decrease in muscle glycogen (Fig 2D).

**Fig 3:**
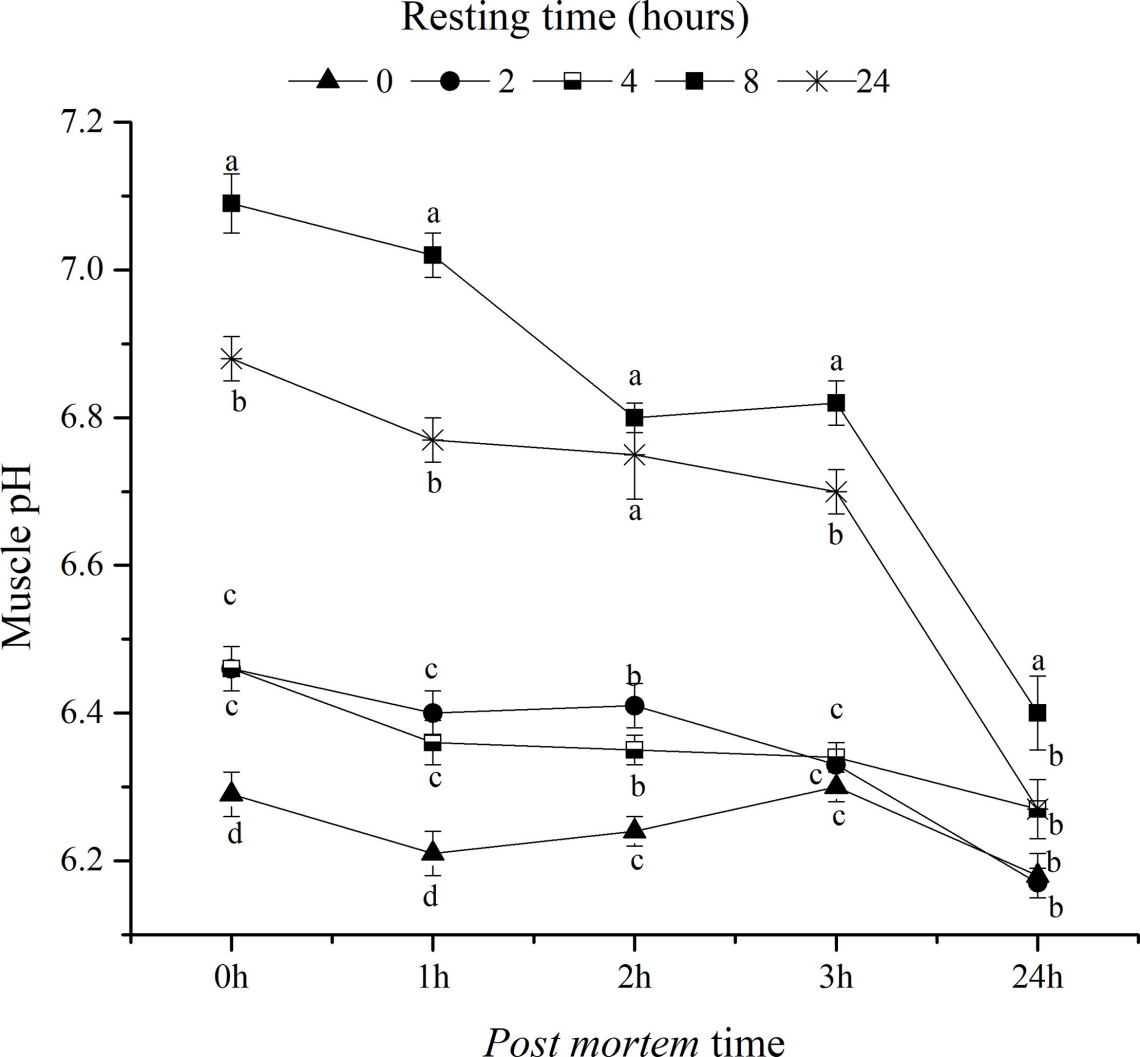
pH evaluation of surubim fillets measured immediately after slaughter and 1, 2, 3 and 24 hours after the different resting times in a holding tank. The bars and error bars denote mean and standard error of means. Different letters in the same resting time of pH evaluation indicate statistical difference (P<0.05) by the Tukey test.

Assessing the pH evolution of the fillets (Fig 3), it was observed that fish submitted to longer resting times (8 and 24 hours) resulted in fillets with higher pH (P <0.05) compared to the others. Surubim slaughtered immediately after transport (0 h of rest) had fillets with initial pH significantly lower (0 and 1 hour post mortem) than the others. However, in the analysis of the fillets 24 hours after slaughter, only the resting time of 8 hours provided fillets with a higher pH.

**Fig 4:**
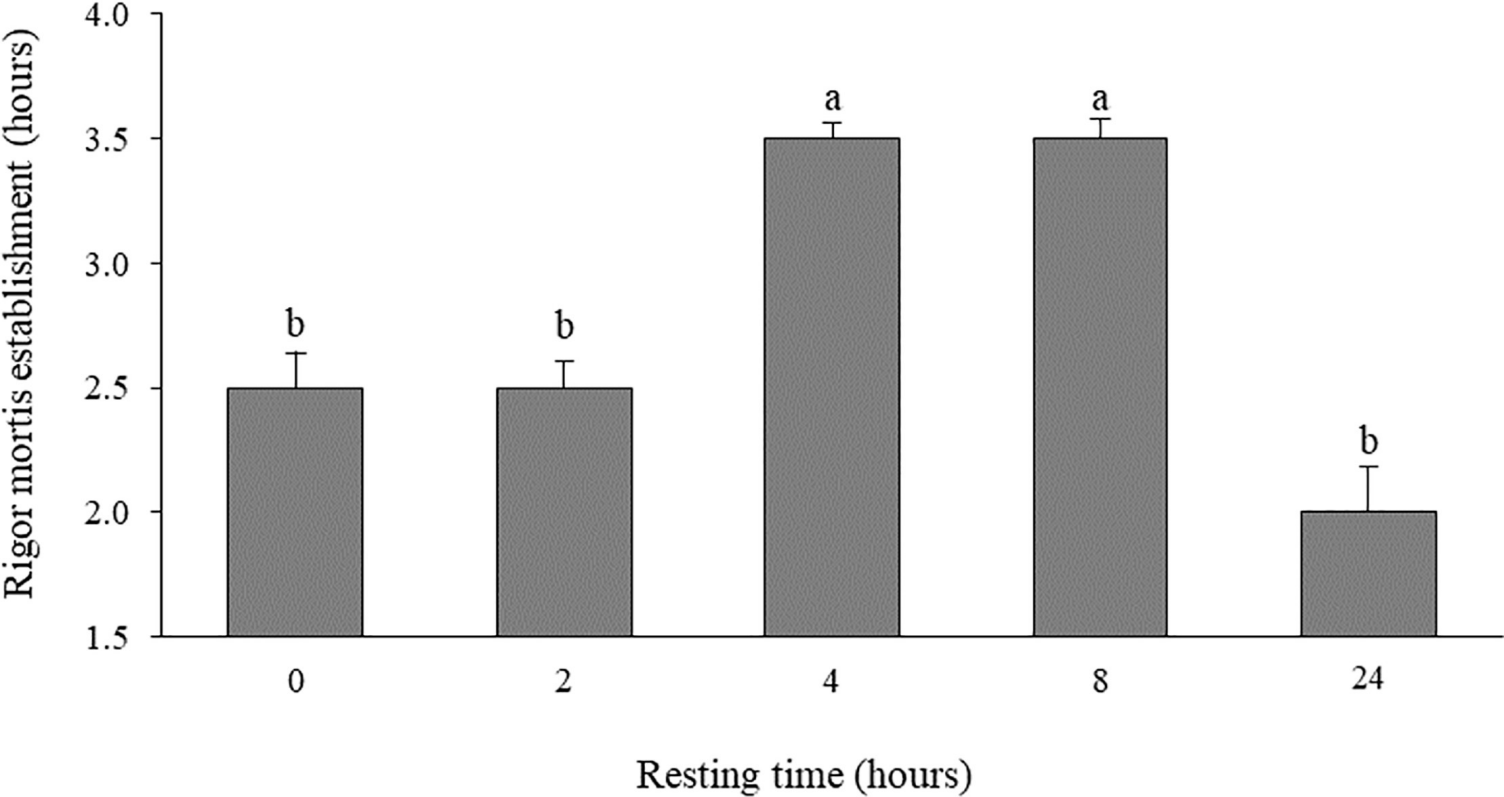
Mean values (± SD) of rigor mortis measurements (tail drop test) for surubim *Pseudoplatystoma* spp. (n = 5) submitted to a different resting time in a holding tank before slaughter. Different letters indicate statistical difference (P<0.05) by Scott-Knott test.

**Fig 5:**
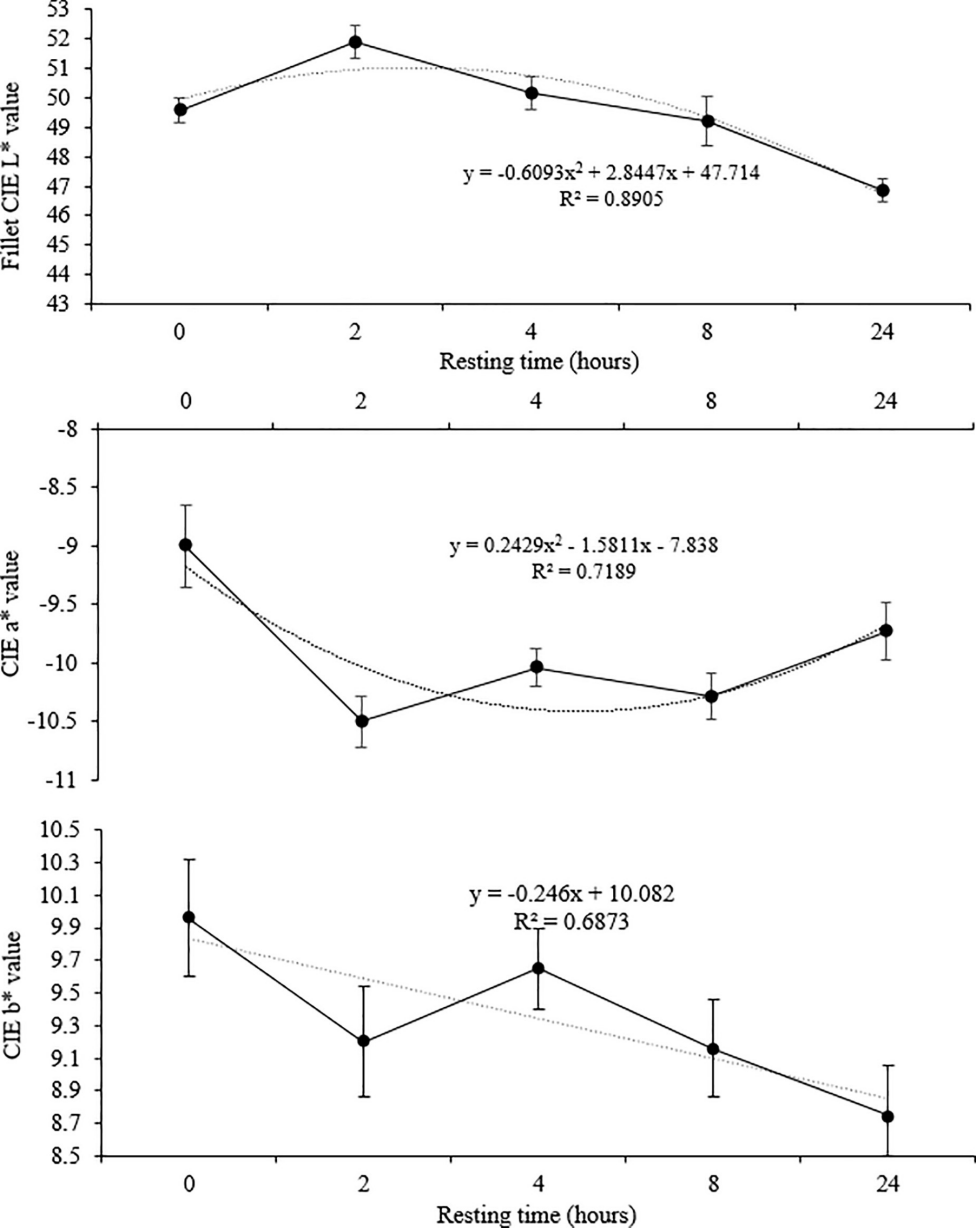
Lightness (CIE L*), yellowness (CIE b*) and redness (CIE a*) of surubim fillets submitted to a different resting time in a holding tank before slaughter. Vertical bars indicate mean standard error.

The rigor mortis establishment time was shorter for the first 2 hours and for 24 hours of resting time, in relation to the times of 4 and 8 hours (Fig 4). Quadratic regressions (P <0.05) were used to compare the color of the fillets for the lightness (CIE L*) and the CIE a* (Fig 5). The highest lightness and the lowest intensity of red (CIE a*) were observed in the fillets of surubim submitted to 2 hours of rest. There was a linear decrease in lightness and an increase in the intensity of red up to 24 hours of rest with the increase in resting times. In CIE b *, a linear decrease (P <0.05) of the yellow intensity of the fillets was observed, as the surubins' resting time increases.

For the parameters of myofibrillar fragmentation index (MFI) and water holding capacity (WHC) of the fillets, there were no significant effect on the different resting times (Table 2).

**Table 2:** Myofibrillar Fragmentation Index (MFI), and Water Holding Capacity (WHC) of surubim fillets submitted to different resting times in a holding tank before slaughter.

Parameters	Resting time in a holding tank (hours)				
	0	2	4	8	24
MFI	67.27 ± 3.63	67.15 ± 1.62	75.80 ± 2.44	76.74 ± 5.71	71.69 ± 3.79
WHC	59.95 ± 0.48	60.37 ± 0.69	61.99 ± 0.78	62.01 ± 0.58	60.62 ± 0.80

Average values ± standard error. Means in the same row with different letters indicate statistical difference by Tukey test (P<0,05).

## Discussion

High stocking densities are a well-known stressor for fish and can induce various physiological changes to the organisms, depending on its severity and duration [29]. In general, serum cortisol levels increase rapidly in response to stress [30]. However, one of the main metabolic responses to stress in fish is an increase in glucose production [31]. Serum glucose levels increase considerably after exposure to an acute stressor and homeostasis is restored within a few hours. Due to the increase in energy metabolism, there is an increase in plasma glucose, which makes this parameter widely used as an indicator of stress in studies with fish [32]. In this study the cortisol level was not an indicator in the dynamics of restoring homeostasis expected in pre-slaughter management.

Different types of acute stress can cause different responses, which can block the release of the steroid. As a result of the type of stress applied, negative "feedback" can occur and in new applications of the stressor, there could be a block in the release of cortisol [33]. This justifies that there was no significant difference in this parameter.

For surubim the demand for glucose was supplied by the breakdown of muscle glycogen. Acute hyperglycemic response has been reported for fish submitted to exhaustive swimming [3] and environmental hypoxia [34]; [35]. Up to 4 hours of resting time, we observed an increase in glucose levels that evidenced the stress profile, which justifies hemoconcentration and therefore changes the hematocrit and MCV levels. The increase in hematocrit right after transport indicates the occurrence of hemoconcentration, this is due to the increased demand for oxygen to maximize the use of the energy substrate in response to the stressor stimulus [36]; [37]. Whereas the increase in MCV immediately after transport is associated with the mobilization of catecholamines released into the bloodstream, indicating osmoregulatory disorders, leading to hemoconcentration as was also observed for hematocrit [38]; [36]; [39]. On the other hand, the decrease in hematocrit and MCV variables after 8h of rest indicates the reestablishment of homeostasis. According to [35] the MCV is used to indicate the osmoregulatory state and is directly involved with cardiac dynamics and blood flow, which support our respiratory dynamics results.

In general, blood gas pressures (pO_2_ and pCO_2_) tend to show little variation in most situations since the first homeostasis mechanism is related to increased heart rate [40]. In this study, this mechanism was not enough to maintain oxygen dynamics, which resulted in acidosis-induced hypoxemia [40]. The increase in the concentration of CO_2_ in the blood is capable of triggering cardiorespiratory responses directly from the interaction of specific CO_2_/pH chemoreceptors, independent of the O_2_ concentration [41].

It is known that an increase in CO_2_ pressure and a concomitant decrease in pH, are inevitable during hypercarbia, a mechanism used to alter the diffusion gradient between blood and water by decreasing pCO_2_ [42]. It is emphasized that the resting time is effective for restoring homeostasis, resulting in blood pH values close to neutrality.

Before slaughtering fish, stress can result in a faster decrease in muscle pH [43]; [44]; [45]. This happens during vigorous exercise, the consumption of adenosine triphosphate (ATP) leading to the anaerobic use of glycogen to replace energy reserves in the muscle [46]. This process persists after death, with glycolysis, resulting in the accumulation of lactic acid in muscle tissues, which, in turn, decreases muscle pH [45]; [12].

Furthermore, fish with a high content of muscle glycogen subjected to pre-slaughter stress can develop fillets with lower pH due to the greater activity of glycolysis in anaerobic conditions. This evidence was confirmed in the present study, where fish subjected to rest for 0 and 2 hours presented higher muscle glycogen, higher plasma glucose and a lower pH of the fillets. The 8-hour resting time was sufficient to reestablish the homeostasis of the fish, which provided a higher final pH of the fillets compared to the other resting times.

Like the muscle glycogen, the hepatic glycogen shows a gradual reduction with the increasing on resting time; however, no statistical difference was found on this parameter. The decrease in hepatic glycogen reveals that it is possible that the metabolite was used as a substrate for energy generation in response to the stress stimulus. This occurred due to the maintenance of glucose metabolism in these animals during the experimental period. This dynamic of use of hepatic glycogen is reported for fish subjected to transport stress [47] and to sustained exercise [48].

Muscle pH affects the appearance of rigor mortis in fish [46]. Several studies show a relationship between low pH and faster onset of rigor in fish [43]; [44]; [45]. The onset of rigor mortis is closely linked to the depletion of ATP and glycogen, which is, the postmortem energy state in the muscle [49]; [9]. Actin and myosin combine to form the actomyosin complex, promoting irreversible muscle contraction, initiating the state of rigor mortis [50]. Thus, pre-slaughter stress can influence the time of entry into rigor. In the present study, the entry of fish in rigor mortis was different. The shorter resting times (0 and 2 hours, more stressed fish) resulted in a faster establishment of rigor mortis compared to fish kept in rest for 4 and 8 hours. This faster entry into rigor mortis is detrimental to the processing industry, since filleting the fish in the state of full rigor leads to a reduction in fillet yield, as well as a decrease of freshness beginning in the post-rigor stage [8]. Therefore, the extension of the pre-rigor period is considered an important factor to maximize the yield of the fillet and its shelf life [51]; [2].

In the present study, we were unable to identify the relationship between minimum pH and the onset of rigor mortis. Fish subjected to 4 and 8 hours of rest entered rigor mortis 3.5 hours after slaughter and these animals had muscle pH different in the measurement performed 3 hours after slaughter (6.34 and 6.82 for fish subjected to 4 and 8 hours of rest, respectively).

In addition, the minimum muscle pH for all treatments was observed only 24 hours after slaughter. It is known that the rigor mortis development is the result of a complex combination of the biochemical processes in the muscle [52]. Although for several species it is observed that the muscle goes into rigor when the pH reaches the minimum, in general terms, there is no constant pH for the muscle to become rigid [53]. A study by [54] demonstrated that pH measurements alone cannot be used to indicate the resolution of the rigor. Apparently, the onset of rigor mortis is a consequence of the early lack of ATP, which is the most visible stress index for establishing rigor mortis [55]. Likewise, a study carried out with rainbow trout (*Oncorhynchus mykiss*) also found no relationship between muscle pH and entry into rigor mortis [56].

It is interesting to note that the resting time of surubim for 24 hours was also detrimental to the onset of rigor mortis, since these animals, despite having the lowest plasma glucose values, entered rigor mortis more rapidly than the others. The intense activity for a long period before slaughter causes the fish to suffer and can completely deplete its glycogen reserves [4]. The high consumption of glycogen due to stress and the simultaneous removal of lactic acid by the circulatory system in the live animal would leave it without glycogen reserves. After death, rigor mortis continues without the production of lactic acid (pH remains high), resulting in fast pre-rigor and total rigor without decreasing the pH, called alkaline rigor mortis [53].

The rapid reduction in post-mortem pH can also lead to a denaturation of muscle proteins, resulting in less water holding capacity and greater drip losses [8], which can also lead to faster muscle softening, which is not beneficial for fish muscle [57]. Nevertheless, in this study, the lower initial pH was not sufficient to affect the water holding capacity and the myofibrillar fragmentation index of surubins. However, it can be inferred that there was a certain degree of protein denaturation, since there were changes in the color of the fillets. It is known that the greater lightness of meat in animals subjected to stress before slaughter can be attributed to the denaturation of sarcoplasmic proteins, which increases the dispersion of light in the muscle [58]. In this study, it is possible to observe a relationship between the lowest initial pH values of fillets with greater lightness, less intensity of red and greater intensity of the yellow color. A previous study showed that these changes in color are related to fish fillets that went through greater acute stress in the pre-slaughter phase [4].

## Conclusion

The resting time of 4 to 8 hours is effective to reestablish homeostasis after transporting surubim, which provides fillets with higher quality and greater length of the pre-rigor mortis period.
